# In vitro prediction of organ toxicity: the challenges of scaling and secondary mechanisms of toxicity

**DOI:** 10.1007/s00204-020-02669-7

**Published:** 2020-02-17

**Authors:** Jan G. Hengstler, Anna-Karin Sjögren, Daniele Zink, Jorrit J. Hornberg

**Affiliations:** 1 IfADo at TU Dortmund, Dortmund, Germany; 2 Cardiovascular, Renal and Metabolism Safety, Clinical Pharmacology and Safety Sciences, R&D, AstraZeneca, Gothenburg, Sweden; 3 NanoBio Lab and Innovations in Food and Chemical Safety Programme, Agency for Science, Technology and Research, Singapore, Singapore; 4 Respiratory, Inflammation and Autoimmunity Safety, Clinical Pharmacology and Safety Sciences, R&D, AstraZeneca, Gothenburg, Sweden

Recently, a study about in vitro prediction of nephrotoxicity has been published (Sjögren et al. 2018). The authors aimed to develop a first-line screening assay that can be applied to guide chemical design in early drug discovery (Johansson et al. 2019). They used a proximal tubular epithelial cell line expressing the organic anion transporter 1 (ciPTEC-OAT1). Renal proximal tubular epithelial cells (PTEC) are frequently affected by compound-induced nephrotoxic effects and are, therefore, widely applied in kidney-specific in vitro methods (Tiong et al. 2014). ciPTEC-OAT1 were incubated with 38 compounds known to be nephrotoxic in humans while 24 were non-nephrotoxic (Sjögren et al. 2018). The assay was performed in a 96-well plate format to facilitate the screening throughput required in drug discovery programs. After an incubation period with test substances for 48 h several fluorophores were added: Hoechst to stain nuclei, LysoTrackerGreen for visualization of lysosomes, MitoTracker Orange to image mitochondria, TOTO-3 as a dead cell indicator and Phalloidin to stain actin cytoskeleton. Subsequently, the cells were fixed by paraformaldehyde and, after high-content imaging, 200 cellular phenotypic parameters were analyzed. The most predictive phenotypic parameters were identified using a machine learning approach by mathematical feature selection algorithms and related to changes of the actin cytoskeleton, mitochondria, nuclear morphology and integrity of the cell membrane (Sjögren et al. 2018). The 62 compounds were tested over a range of six concentrations covering the maximal plasma concentration (*C*_max_) of the individual drugs. The authors evaluated at what concentration range, in relation to the *C*_max_, the assay delivered optimal predictivity. They found that whereas an increasing number of toxic compounds were identified as the test concentration increased, there were no false positives in the assay up to a test concentration of 200-fold the *C*_max_. Overall, a sensitivity of 66% and a specificity of 100% were reported.

The study (Sjögren et al. 2018) initiated a discussion with further scientists working with in vitro tests to predict organ toxicity (Zink 2019; Sjögren and Hornberg 2019), which led to a consensus concerning two aspects that may be of general interest: first, the necessity to use higher concentrations in the culture medium compared to blood concentrations in vivo; second, the need to improve our possibilities to predict toxicity caused by ‘secondary mechanisms’.

## The use of high in vitro concentrations compared to human ***C***_max_ in plasma

A frequently used strategy to predict organ toxicity in vitro is to use a validation set of compounds known to cause an increased risk of organ toxicity for a certain dosing schedule (positive reference compounds) and compounds that do not lead to an increased risk (negative reference compounds). A compound tested in vitro at a specific concentration either leads to a positive or negative test result. The in vitro test is typically performed at a concentration range around and above the plasma peak concentrations (*C*_max_) in humans. By comparison of the in vitro test result (positive, negative) to the clinical safety profile (toxic, non-toxic), statistical performance metrics, such as sensitivity, specificity or accuracy can be calculated. Importantly, the use of relatively high concentrations, often 20- to 200-fold higher than the therapeutic *C*_max_, leads to a better accuracy than lower concentrations. Also in the present study (Sjögren et al. 2018), while most toxic drugs were identified at concentrations around or below 20-fold the therapeutic *C*_max_, testing at higher concentrations (up to 200-fold the *C*_max_) achieved a very good sensitivity and specificity. It is relevant to note that, for screening assays that are to be applied during early drug discovery, it is essential to maintain high specificity, to avoid deselection of potentially promising compound series (whereas high sensitivity becomes more relevant towards selection of actual candidate drugs). Therefore, Sjögren et al. suggested a threshold of 200-fold, since beyond that threshold false positives were observed. Still, this leads to the question, whether such high concentrations are still relevant for the human in vivo situation. From a cell biology point of view this objection is more than justified. Nevertheless, the ultimately critical criterion is if the test condition leads to a correct prediction of the clinical situation or not. Also in previous studies on hepatotoxicity (O’Brien et al. 2006; Persson et al. 2013; Albrecht et al. 2019), a similar observation was made that higher concentrations than *C*_max_ better differentiated between positive and negative control compounds. There are several potential explanations for this. For example, toxicity is clinically often observed after chronic exposure and accumulation of subtle insults, while tests in the lab usually rely on more dramatic insults upon short-term incubation. While this is certainly correct, it may not be sufficient to explain the situation. A recent study with primary human hepatocytes incubated 30 test compounds for 1, 2 and 7 days and analyzed cytotoxicity (Gu et al. 2018). As expected, longer incubation periods led to lower EC_50_ values. However, this decrease of EC_50_ values after 7 days compared to shorter incubations occurred also for negative test compounds and did not allow a better differentiation between positive and negative controls by just using longer incubation periods (Albrecht et al. 2019). This further exemplifies that it is relevant to maintain a low false positive rate.

Another potential reason for the high concentrations required in vitro may be that target organs may in reality experience much higher concentrations in certain cell compartments than indicated by measurements of the plasma *C*_max_, or compounds require active transport and/or metabolism for the toxicity to become apparent (Will and Dykens 2014). Down-regulation of transporters for drugs and other xenobiotics and of drug-metabolizing enzymes applies to PTEC (Jenkinson et al. 2012; Lash et al. 2008; Tiong et al. 2014) as well as to hepatocytes (Gomez-Lechon et al. 2014; Vildhede et al. 2015; Vinken and Hengstler 2018). In fact, although lots of efforts are spent currently on the development of improved cell models, there is no PTEC or hepatocyte model available that would in vitro fully recapitulate the in vivo performance, in particular with respect to the quantitative expression levels of transporters and drug-metabolizing enzymes. In light of these differences it would be rather surprising if cells in vitro would show the same concentration response as in vivo. Apart from developing improved cell models, the exploration of scaling factors that account for different transporter and enzyme expression levels in vitro and in vivo may be useful. Such scaling factors have been successfully applied recently in physiologically based modelling of drug clearance (Chan et al. 2019; Vildhede et al. 2015).

Down-regulation of drug-metabolizing enzymes in vitro may in part explain why ifosfamide was not detected as toxic in the present study (Sjögren et al. 2018), because ifosfamide requires metabolic activation by cytochrome P450 enzymes. However, a more likely explanation is that this false negative is a result of the fact that the set of endpoints analyzed in the high content imaging assay did not reflect the mechanism of toxicity for ifosfamide: the compound does induce gene expression of the oxidative stress marker HMOX1 in ciPTEC-OAT1. Apart from other potential solutions as discussed above, this limitation may be overcome by the additional inclusion of a drug metabolizing system into the test in future (Godoy et al. 2013).

## The challenge of ‘secondary mechanisms’ of organ toxicity

Some cell types are particularly susceptible to the toxic effects of chemicals, such as PTEC of the kidney or hepatocytes of the liver. A relatively large fraction of nephrotoxic or hepatotoxic compounds act by causing damage directly to these cell types. However, adverse effects on the kidney or liver can also be induced by mechanisms that initially do not involve PTEC or hepatocytes. Examples are altered hemodynamics or crystal formation (kidney) as well as compromised cholangiocytes or obstructions of the biliary tract (liver). Here we refer to that as mechanisms of secondary toxicity. Of course, identification of compounds acting by primary mechanisms represents an important milestone. Nevertheless, the final goal is to identify all toxic compounds, independent of their mechanism of action. A practical implication for in vitro testing is that tests for primary mechanisms, e.g. based on cultivated PTEC or hepatocytes, should be accompanied by a battery of tests that detect the secondary mechanisms. However, relatively little is known about how accurately these secondary toxicities can be identified.

In the field of nephrotoxicity, a categorization with three sub-groups of compounds has been introduced (Li et al. 2013, 2014; Kandasamy et al. 2015; Su et al. 2016).

*Group 1* Nephrotoxicants that damage PTEC.

*Group 2* Nephrotoxicants that damage the kidney by other mechanisms, e.g. by altering renal hemodynamics, or by crystal formation; examples are indinavir, which exerts nephrotoxicity because of crystallization and stone formation; lisinopril, an angiotensin-converting enzyme inhibitor which causes nephrotoxicity by hemodynamic effects.

*Group 3* Non-nephrotoxic compounds, i.e. compounds that up to a known *C*_max_ do not cause nephrotoxicity in humans.

To test the hypothesis that the assay would be more sensitive to drugs that directly damage PTEC, the present study of Sjögren and colleagues (2018) also analyzed the predictive performance for those drugs separately (i.e. compared group 1 and group 3). This resulted in a performance metrics of 75% (15 of 20 drugs) for sensitivity and 100% for specificity (24 of 24 drugs).

The situation becomes more complex for the group 2 compounds: of the 18 compounds in that category, 10 tested positive and 8 negative (Sjögren et al. 2018). Previous studies observed a relatively high percentage of negative results for group 2 compounds up to very high concentrations of 1000 µg/ml (Li et al. 2013, 2014; Su et al. 2016). Differences in outcomes between the studies may relate to the analyzed compounds and the endpoints. In the future, it will be important to identify and study larger numbers of group 2 compounds. These studies should include the currently available PTEC-based tests to allow a direct comparison; moreover, in vitro tests of secondary mechanisms of nephrotoxicity, e.g. precipitation tests and tests for vascular dysfunction should be included. These studies should show with which sensitivity and specificity group 2 compounds can be identified.

Currently, much effort is invested in the development of in vitro systems (Sachinidis et al. 2019; Leist et al. 2017) particularly to predict nephrotoxicity (Adler et al. 2016; Li et al. 2013, 2014; Su et al. 2016; Kandasamy et al. 2015), hepatotoxicity (Godoy et al. 2015, 2016; Proctor et al. 2017; Grinberg et al. 2014), cardiotoxicity (Archer et al. 2018; Sampaio et al. 2016; Reis-Mendes et al. 2017; Chaudhuri et al. 2016), developmental toxicity (Waldmann et al. 2014, 2017), and the improvement of physiologically based pharmacokinetic modeling and other modeling strategies (Ghallab et al. 2016; Chan et al. 2019). The results of the here discussed high content screening assay (Sjögren et al. 2018) show that it is possible to implement a high-throughput screening assay in early drug discovery to guide chemical design away from nephrotoxicity. Compounds acting via damaging PTEC can be identified with a relatively high accuracy, and further studies are needed to clarify whether addition of further readouts to a test battery improves the identification or mechanistic investigation of compounds acting by secondary mechanisms. In vitro research in different areas, nephrotoxicity, hepatotoxicity, cardiotoxicity, neurotoxicity and developmental toxicity, seems to be confronted with similar challenges.
