# Correction to: In vitro prediction of organ toxicity: the challenges of scaling and secondary mechanisms of toxicity

**DOI:** 10.1007/s00204-021-03090-4

**Published:** 2021-07-20

**Authors:** Jan G. Hengstler, Anna-Karin Sjögren, Daniele Zink, Jorrit J. Hornberg

**Affiliations:** 1 IfADo at TU Dortmund, Dortmund, Germany; 2 Cardiovascular, Renal and Metabolism Safety, Clinical Pharmacology and Safety Sciences, R&D, AstraZeneca, Gothenburg, Sweden; 3 NanoBio Lab and Innovations in Food and Chemical Safety Programme, Agency for Science, Technology and Research, Singapore, Singapore; 4 Respiratory, Inflammation and Autoimmunity Safety, Clinical Pharmacology and Safety Sciences, R&D, AstraZeneca, Gothenburg, Sweden

## Correction to: Archives of Toxicology (2020) 94:353–356 10.1007/s00204-020-02669-7

The article “In vitro prediction of organ toxicity: the challenges of scaling and secondary mechanisms of toxicity”, written by Jan G. Hengstler, Anna-Karin Sjögren, Daniele Zink and Jorrit J. Hornberg, was originally published Online First without Open Access. After publication in volume 94, issue 2, pages 353–356 the author decided to opt for Open Choice and to make the article an Open Access publication. Therefore, the copyright of the article has been changed to © The Author(s) 2020 and the article is forthwith distributed under the terms of the Creative Commons Attribution 4.0 International License, which permits use, sharing, adaptation, distribution and reproduction in any medium or format, as long as you give appropriate credit to the original author(s) and the source, provide a link to the Creative Commons licence, and indicate if changes were made. The images or other third party material in this article are included in the article’s Creative Commons licence, unless indicated otherwise in a credit line to the material. If material is not included in the article’s Creative Commons licence and your intended use is not permitted by statutory regulation or exceeds the permitted use, you will need to obtain permission directly from the copyright holder. To view a copy of this licence, visit http://creativecommons.org/licenses/by/4.0.

Open access funding enabled and organized by Projekt DEAL.

Original article has been corrected.

